# Alcohol or Opium?

**Published:** 1897-12

**Authors:** F. A. Schmidt

**Affiliations:** LaGrange, Tex.


### Correspondence.

### Alcohol or Opium?

LaGrange, Tex., Nov. 17, 1897.

*Editor Texas Medical Journal:*

I have read with a great deal of interest your remarks concerning Dr. Guiteras, every word of which I heartily approve; Your address before the Woman's Christian Temperance Union of Texas is very interesting and instructive reading. There is many a truth in it, but also many a point where people differ with you, nevertheless they may have the good of the human race at heart as you do, as have also the ladies whom you addressed.

I, for instance, take issue with you in regard to real temperance, if as is generally the case, this word is used where total abstinence is meant; and I would even agree with you in this (total abstinence) if possibly it could be realized in the future. Many centuries have passed since fabulous Noah imbibed his home-made beverage, and as many a reformer during these ages has tried to shut off the poison alcohol altogether from unsuspecting mankind, without in the least, diminishing its use. Would it not be the better way to lay total abstinence aside, and to plead, urge and practice real temperance? That is, the moderate use of the article?

What great benefit besides would a country derive from the universal use of mild beverages--such as wine or beer—instead of whiskey which, as if by common consent is considered our national drink? Other countries thrive on this plan, why not we?

My experience as a physician taught me, that the mere *pleading* of total abstinence is peculiarly "conducive" to the use of worse narcotics, such as morphine, cocaine, and within late years of the "hashish" of the Turk, who in a late medical journal is lauded for his abstinence of alcohol—(for the sake of Indian hemp).

Within a period in my recollection as a pharmacist and physician of forty-seven years, the opium habit, especially among women, which in early days was confined to a few of the lower classes, has so alarmingly increased, and has invaded our best classes of society to a degree, as to become a great danger to society, and as to leave me in doubt which of these vices exerts the most baneful influence upon our people and posterity: The abuse of alcohol by our men, or the indiscriminate use of morphine, cocaine and allied drugs by a daily increasing number of our women—and men for that matter.

*I may be mistaken, but from close personal observation, I can not help coming to the conclusion that the increasing popularity of baneful narcotics is in exact ratio to the agitation of total abstinence;* not temperance, mind you.

Respectfully,

F. A. Schmidt, M. D.

